# A new M23-based ELISA assay for anti-aquaporin 4 autoantibodies: diagnostic accuracy and clinical correlation

**DOI:** 10.1186/s13317-019-0115-7

**Published:** 2019-06-19

**Authors:** Marilina Tampoia, Letizia Abbracciavento, Giuseppina Barberio, Martina Fabris, Nicola Bizzaro, Study Group on Autoimmune Diseases of the Italian Society of Clinical Pathology and Laboratory Medicine, Italy

**Affiliations:** 1 Clinical Pathology Laboratory, Polyclinic of Bari, Department of Biomedical Sciences and Human Oncology, University of Bari, Piazza Giulio Cesare 11, 70124 Bari, Italy; 2 Laboratory Medicine, Department of Clinical Pathology, Treviso Hospital, Treviso, Italy; 3 Laboratory of Immunopathology and Allergology, University Hospital Udine, P.le S. Maria della Misericordia 15, 33100 Udine, Italy; 4 Laboratory of Clinical Pathology, San Antonio Hospital, Azienda Sanitaria Universitaria Integrata di Udine, Udine, Italy

**Keywords:** Anti-AQP4 autoantibodies, NMO spectrum disorders, Enzyme-linked immunosorbent assay, Analytical imprecision

## Abstract

**Purpose:**

Although many assays have been developed to detect anti-aquaporin-4 (AQP4) antibodies, most of these assays require sophisticated techniques and are thus only available at specialized laboratories. The aim of this study was to evaluate the analytical and clinical performance of a new commercial enzyme-linked immunosorbent assay (ELISA RSR, AQP4 Ab Version 2) to detect anti-AQP4 antibodies performed on a fully automated system (SkyLAB 752).

**Methods:**

Serum samples from 64 patients with neuromyelitis optica spectrum disorders (NMOSD) (including NMO, longitudinally extensive myelitis-LETM, optical neuritis and myelitis) and 27 controls were tested for anti-AQP4 antibodies. All sera were previously tested using an indirect immunofluorescence (IIF) method on primate tissue, as the reference method. Commercial control sera were used to determine within-run, between-day and within-laboratory precision (CLSI guidelines).

**Results:**

At a cut-off value of 2.1 U/mL as determined by ROC curves, sensitivity and specificity for NMO were 83.3% and 100%, respectively. The ELISA assay provided 100% concordant results with the reference IIF method. The median concentration of anti-AQP4 antibodies was statistically higher in patients with NMO than in patients with LETM (*p *= 0.0006) or with other NMOSD and in controls (*p *< 0.0001). At the concentration of 12.4 and 28.1 U/mL, the within-run, between-day and within-laboratory coefficients of variation (CV) were 3.2% and 3%, 7.6% and 7.4%, and 8.2% and 8.0%, respectively.

**Conclusions:**

This new ELISA method performed on a fully automated system, showed high sensitivity and absolute specificity, good CV in precision tests, and provided observer-independent quantitative results.

## Introduction

Neuromyelitis optica (NMO) is an autoimmune demyelinating disease of the central nervous system that predominantly targets the optic nerve and the spinal cord, and it is characterized by the presence of anti-aquaporin 4 antibodies in the patient serum [1].

The clinical spectrum of NMO as defined by Wingerchuk et al. [2] comprises cases of optic neuritis (ON) and myelitis that occur simultaneously, cases in which the two index events do not develop simultaneously but successively, and cases of limited or inaugural forms, such as single or recurrent events of longitudinally extensive myelitis (LETM) or recurrent ON [2–6]. More rarely, patients may present with brain stem encephalitis [7, 8].

In 2004, the Mayo Clinic group for the first time found a specific NMO-immunoglobulin G (IgG) in the sera of NMO patients, which binds at or near the blood–brain barrier in the mouse brain [9].

In 2005, the target epitope of NMO-IgG was identified as aquaporin-4 (AQP4), a water channel densely expressed in astrocytic foot processes at the blood–brain barrier [10].

AQP4 is present in two major isoforms, the full-length 323 amino acid M1 isoform with translation initiation at Met-1 and the shorter 301 amino acid M23 isoform with translation initiation at Met-23. The two isoforms differ by 22 N-terminal cytosolic amino acids, which although are not a target of AQP4 antibodies, influence the quaternary structure of the protein on the cell surface [11]. Both isoforms are assembled into homo- and heteromeric AQP4 tetramers; only the M23 isoform assembles in membranes as regular square arrays, called orthogonal arrays of particles (OAP) [12].

In 2006, the positive identification of serum anti-AQP4 antibodies has been included in the revised diagnostic criteria for NMO [13]. Therefore, a test able to detect anti-AQP4 antibodies with high sensitivity and specificity is crucial in clinical practice for the diagnosis and treatment of this neurological disorder.

Since the discovery of anti-AQP4 antibodies, several assays for their detection have been proposed, including indirect immunofluorescence (IIF) on various frozen sections from mouse, rat, or non-human primate tissue; IIF on transfected cells expressing human AQP4, quantified either visually by fluorescence microscopy [cell-based assay (CBA)] or quantitatively by flow cytometry (FACS); and partially purified AQP4 detected by enzyme-linked immunosorbent assay (ELISA), radioimmunoprecipitation assay (RIPA) and fluorescence immunoprecipitation assay (FIPA). However, the diagnostic accuracy (e.g. sensitivity and specificity) of these methods varies broadly [14]. Numerous technical aspects may potentially impact on assay accuracy, including type and species of the tissue sections used, transfection and fixation methods, AQP4 species and isoforms.

Currently, CBA shows a significantly higher mean diagnostic accuracy than the other methods: 76.5% versus 48.5–62.3% [14]. However, CBA provides only semi-quantitative results, is observer dependent, labor-intensive and time-consuming, especially when titration of sera is requested.

Protein-based assays, such as ELISA, may potentially solve technical problems: are easy to use, allow large-scale analysis and have the potential to be automated.

Hayakawa et al. [15] employed for the first time an ELISA with recombinant rat AQP4 as the coating antigen to directly detect serum anti-AQP4 antibodies. A human AQP4 ELISA yielded similar sensitivity but higher specificity [16]. Shortly thereafter was developed the first commercial ELISA manufactured by RSR Ltd. (Cardiff, UK) using the M1-AQP4 isoform; however, the sensitivity reported in validation studies by Isobe (48.3%) [17], Fryer (58%) [18] and Jarius (75.8%) [19], was too low to represent a valid alternative to CBA.

Recently, a new version of the ELISA-RSR assay has been developed based on the human recombinant AQP4 M23 isoform; the present study aimed to evaluate the analytical and clinical accuracy of this new M23-ELISA performed in fully automation.

## Materials and methods

### Patients

Serum samples from 64 patients with NMO spectrum disorders (NMOSD) (24 with NMO, 24 with LETM, 13 with isolated ON, and three with myelitis) and from 27 controls [17 patients with multiple sclerosis (MS) and 10 healthy subjects (HS)] were collected in four Italian centers: the Laboratory of Clinical Pathology of Bari, Treviso and Udine, and the Regional Referring Multiple Sclerosis Centre (CRESM), University Hospital S. Luigi Gonzaga in Orbassano.

All NMO patients met Wingerchuk’s 2006 criteria. All LETM patients had clinically defined myelitis with spinal cord lesions extending over three or more segments. The diagnosis of MS was made according to McDonald’s criteria.

The same serum samples were previously tested for the presence of NMO-IgG using a multiparametric commercial IIF assay (“Neurology Mosaic 17”, Euroimmun, Luebeck, Germany).

The patients’ demographic and serological data are summarized in Table 1.

**Table 1:** Demographic, clinical and serological features from patients and controls

	NMO spectrum disorders				Controls	
	NMO (n = 24)	LETM (n = 24)	ON (n = 13)	Myelitis (n = 3)	MS (n = 17)	HS (n = 10)
Gender (F/M)	21/3	17/7	8/5	3/0	13/4	7/3
Mean age (range)	50 (29–78)	51 (4–78)	39 (13–55)	52 (46–58)	38.4 (11–77)	39.5 (26–57)
AQP4-Ab (ELISA)						
Median (U/mL) range	27.9 (1.5–464)	1.5 (1.5–214.5)	1.5 (1.5–1.9)	1.5 (1.5–3.1)	1.5 (1.5–1.85)	1.5 (1.5–2.1)
Frequency (%)	20/24 (83.3%)	6/24 (25%)	0/13 (0%)	1/3 (33.3%)	0/17(0%)	0/10 (0%)

### Methods

#### Anti-AQP4 measurement

Anti-AQP4 antibodies were determined using a new commercial ELISA assay (RSR Ltd) coated with the human recombinant M23 isoform of AQP4, on a fully automated ELISA processing system (SkyLAB 752—DASITGroup, Cornaredo, Italy). This ELISA method is based on the ability of autoantibodies to act divalently and form a bridge between AQP4 coated onto plate wells and AQP4-biotin in a liquid phase using purified recombinant human AQP4 preparations.

Briefly, 50 μL of five anti-AQP4 calibrators (1.5, 5, 20, 40 and 80 U/mL), of three controls and of patient samples were distributed into the respective wells. One well was left empty for blank. 25 μL of biotinylated AQP4 was then pipetted into each well (except blank). After incubation for 2 h at room temperature (RT) on a shaker, the wells were washed three times with diluted wash solution. Subsequently, 100 μL of diluted streptavidin–peroxidase was added into each well (except blank). After incubation for 20 min at RT on a shaker, wash steps were repeated. Next, 100 μL of tetramethylbenzidine was pipetted into each well (including blank) and incubated for 20 min in the dark at RT without shaking. Finally, 100 μL stop solution was added to each well (including blank) and the plates were shaken for 5 sec. The absorbance of each well was read at 450 and 405 nm, blanked against a well containing 100 μL of tetramethylbenzidine (TMB) substrate and 100 μL stop solution only. A calibration curve was established by plotting the calibrator concentration on the x-axis (log scale) against the absorbance of the calibrators on the y-axis (linear scale). AQP4 autoantibody concentrations in patient sera were then read off the calibration curve.

Serological analyses were performed blindly and all patient records and information remained anonymous, according to the ethical standards as formulated in the Declaration of Helsinki (Edinburgh 2000).

### Analytical evaluation

#### Precision study

Two commercial positive control sera provided by the manufacturer (anti-AQP4 C-1, 12.1 U/mL and C-2, 28 U/mL), were assayed in triplicate, in five independent runs on different days for within-run, between-day and within-laboratory precision, in accordance with the Clinical and Laboratory Standard Institute (CLSI) EP15-A3 guideline [20].

### Statistical analysis

The ANOVA test was used to calculate within-run, between day and within-laboratory precision.

The optimal anti-AQP4 cut-off value was determined by means of the receiver operating characteristic (ROC) curves [21].

The diagnostic sensitivity of ELISA for anti-AQP4 antibodies was calculated separately in 24 patients with NMO, in 24 patients with LETM and cumulatively in all patients with NMOSD; the specificity was calculated in controls. Clinical utility of ELISA was evaluated by likelihood ratios (LR). Per convention, tests yielding positive LR (pLR) > 10 or negative LR (nLR) < 0.1 are considered clinically useful. The Kruskal–Wallis test was used to evaluate differences in anti-AQP4 antibody levels in patients and in the control group, and the Mann–Whitney unpaired U-test was used to compare the median autoantibody levels between groups. Differences in percentage of anti-AQP4 antibodies between NMO and LETM patients were assessed by the Fisher’s exact test. *P* values < 0.05 were considered to indicate statistical significance.

MedCalc software (Mariakerke, Belgium) was used for ROC curve analysis and all statistical analyses were performed using GraphPad Prism Version 5 and Analyse-it Version 4.10.2.

## Results

### Precision test

The mean concentration of C-1 was 12.41 U/mL, the within-run CV% was 3.2%, the between-day CV% was 7.6% and the within-laboratory CV% was 8.2%.

The mean concentration of C-2 was 28.12 U/mL, the within-run CV% was 3.0%, the between-day CV% was 7.4% and the within-laboratory CV% was 8.0%.

The total repeatability, expressed as standard deviation (SD) (within-laboratory), resulted 1.02 for C-1 and 2.25 for C-2, closed to the verification value (1.96 and 4.20, respectively) obtained on the basis of the repeatability declared by the manufacturer [SD C-1 = 1.30 and SD C-2 = 2.77]. The data on precision study are shown in Table 2.

**Table 2:** Evaluation of anti-AQP4 antibody ELISA assay precision according to CLSI EP15-A

Positive control (C-1) Mean 12.41 U/mL	SD	95% CI	CV (%)
Within-run	0.40	0.28 to 0.71	3.2
Between day	0.94	0.44 to 3.49	7.6
Within laboratory	1.02	0.64 to 2.51	8.2

Positive control (C-2) Mean 28.12 U/mL	SD	95% CI	CV (%)
Within-run	0.83	0.58 to 1.46	3.0
Between day	2.09	1.18 to 6.15	7.4
Within laboratory	2.25	1.45 to 6.21	8.0

*SD* Standard deviation, *95% CI* confidence interval, *CV* coefficient of variation

### Anti-AQP4 measurement

Determination of anti-AQP4 antibodies by ELISA allowed quantitative measurements of antibody levels. In patients with NMOSD, antibody levels varied between 1.5 U/mL and 464 U/mL. The median antibody level was 27.9 U/mL (range, 1.5–464 U/mL) in NMO patients, 1.5 U/mL (range, 1.5–214.5 U/mL) in LETM patients, 1.5 U/mL (range, 1.5–1.9 U/mL) and 1.5 U/mL (range, 1.5–3.1 U/mL) in ON and myelitis patients, respectively. In controls, the mean concentration was 1.5 U/mL (range, 1.5–2.1 U/mL).

**Fig. 1:**
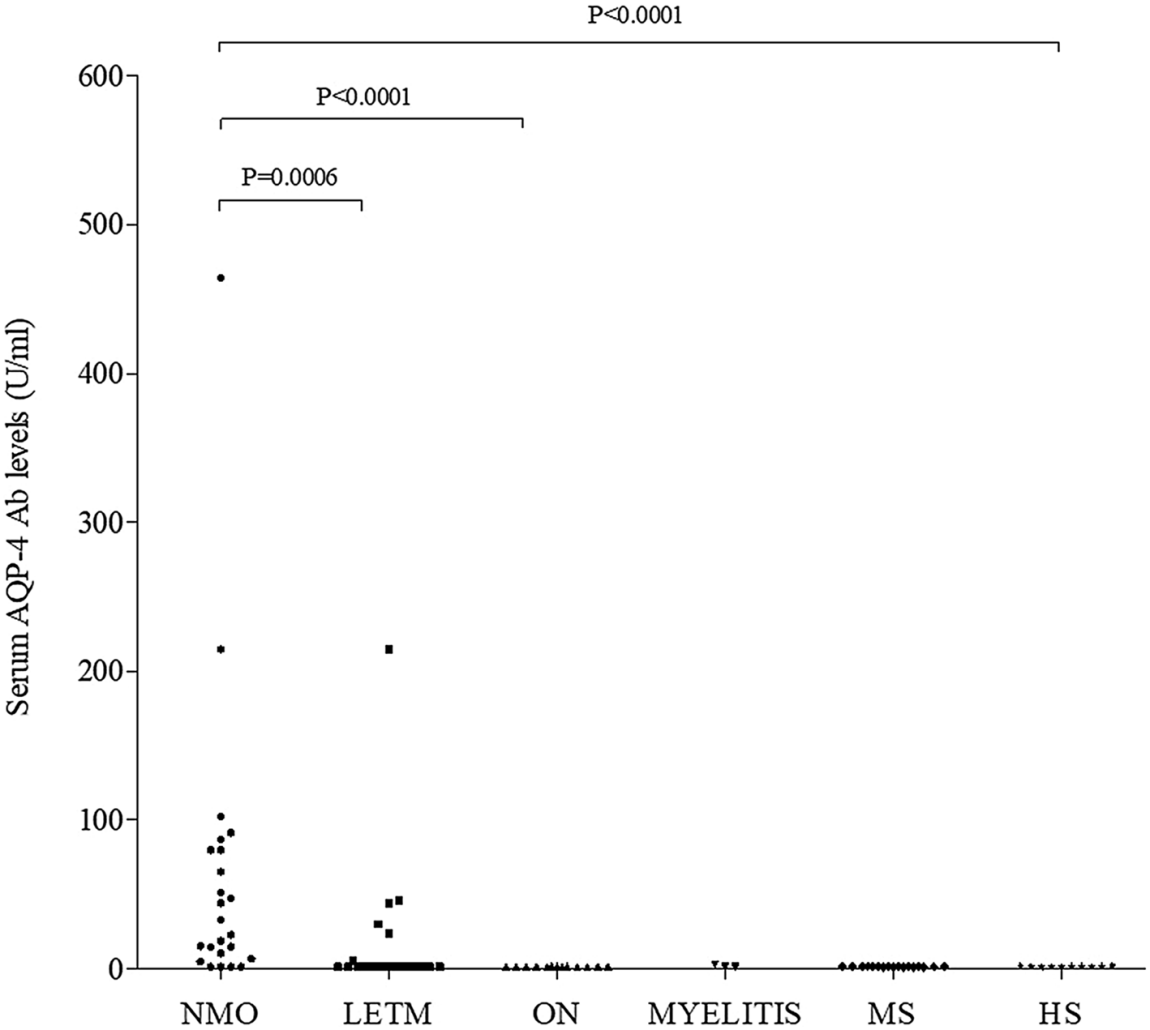
Distribution of anti-AQP4 autoantibody levels, expressed in Units/mL, in patients with neuromyelitis optica (NMO), longitudinally extensive transverse myelitis (LETM), optic neuritis (ON), myelitis and controls, including patients with multiple sclerosis (MS) and healthy subjects (HS). Kruskal Wallis test, *p * < 0.0001; the median level of anti-AQP4 Ab was statistically higher in patients with NMO than in LETM and ON patients (Mann–Whitney Unpaired U-test, *p * = 0.0006 and *p * < 0.0001, respectively)

The median serum level of anti-AQP4 antibodies was higher in patients with NMO than in patients with other NMOSD and in controls (Kruskal–Wallis test, *p *< 0.0001). In particular, the median level of anti-AQP4 antibodies was higher in patients with NMO than in LETM and ON patients (Mann–Whitney Unpaired U-test, *p *= 0.0006 and *p *< 0.0001, respectively) (Fig. 1).

**Fig. 2:**
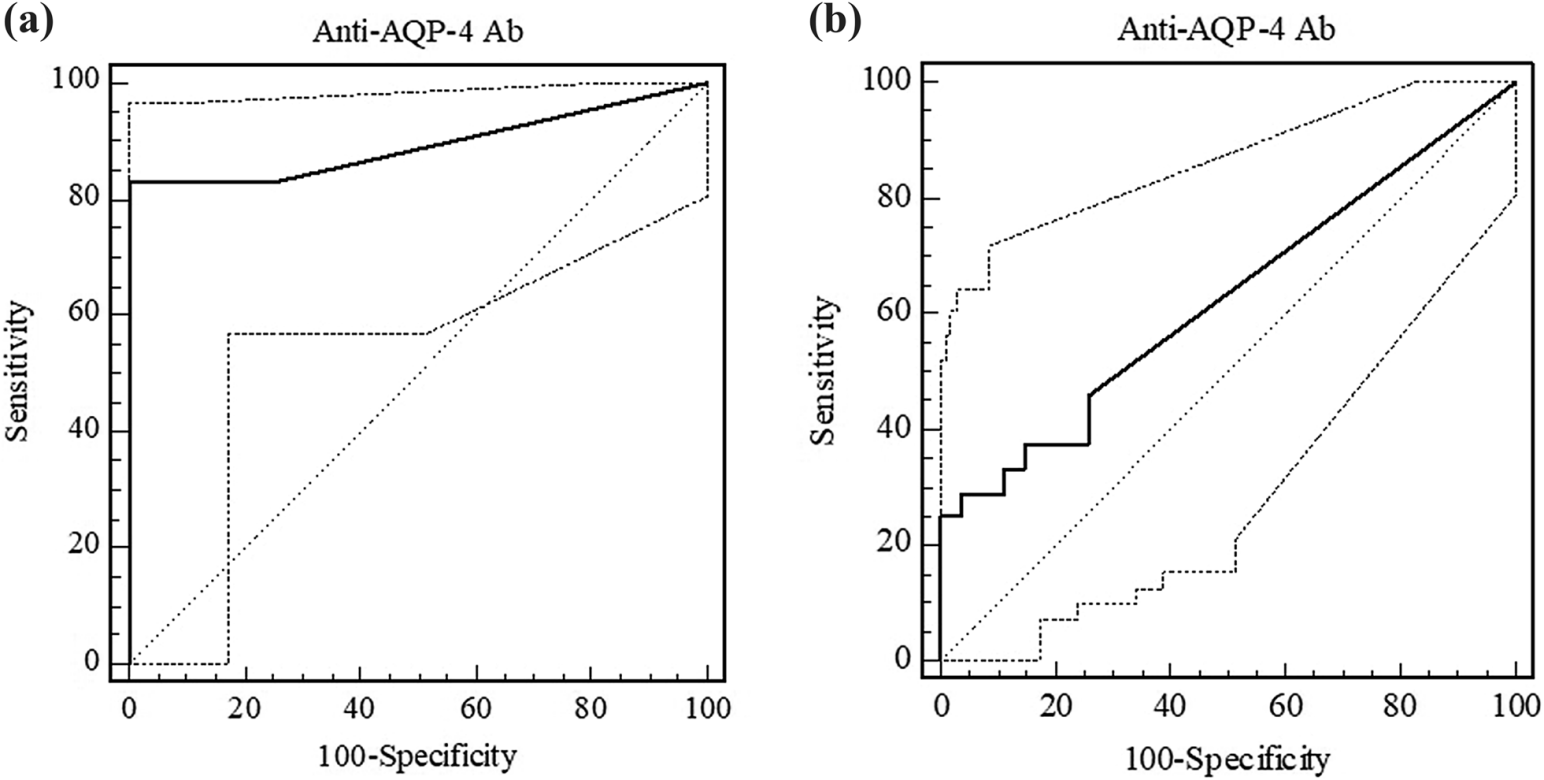
**a** Receiver operating-characteristic (ROC) plot analysis of anti-AQP4 autoantibodies determined by ELISA in patients with NMO. The area under the curve (AUC) is 0.895. **b** ROC plot analysis of anti-AQP4 autoantibodies for LETM. AUC, is 0.625. The continuous line refers to the ELISA method; dotted lines represents 95% confidence interval

At a cut-off value of 2.1 U/mL, as determined by the ROC curve, anti-AQP4 antibody ELISA had a sensitivity of 83.3% (95% CI 62.6–95.2) in patients with NMO, a specificity of 100% (95% CI 87.1–100), a very high positive pLR of ∞ and a nLR of 0.17 (Table 3). The value of the area under the curve (AUC) calculated in patients with NMO, was 0.895 (95% CI 0.777–0.963) (Fig. 2).

**Table 3:** Diagnostic sensitivity, specificity and likelihood ratios (pLR, nLR) in patients with NMO and LETM

	NMO	LETM
Cut off value (U/mL)	> 2.1	> 2.1
Sensitivity (%)	83.3 (CI 62.6–95.2)	25 (CI 9.8–46.7)
Specificity (%)	100 (CI 87.1–100)	100 (CI 87.1–100)
Accuracy^a^	83.3	25
pLR	∞	∞
nLR	0.17	0.75

^a^Accuracy = (Sensitivity × Specificity)/100

At the same cut-off value, sensitivity and specificity in patients with LETM were 25% (95% CI 9.8–46.7) and 100% (95% CI 87.1–100), respectively, pLR was ∞ and nLR was 0.75 (Table 3). The difference in percentage of anti-AQP4 antibodies between NMO patients compared to LETM patients (83.3% and 25%, respectively) was statistically significant (Fisher’s exact test, *p *= 0.0001).

None of patients with ON and one patient with myelitis were positive. All controls were negative for anti-AQP4 antibodies.

The agreement between the multiparametric commercial IIF assay and ELISA was absolute (Cohen’s Kappa = 1.00).

## Discussion

Anti-AQP4 antibodies is an important tool in the diagnostic workup of patients with NMO and have been included in the revised diagnostic criteria for this condition [13]. More recently, anti-AQP4 antibodies have been found also in a subset of patients with isolated transverse myelitis [4], patients with isolated optic neuritis [4–6] and patients with NMOSD and co-existing connective tissue diseases [22–24]. Therefore, the International Panel for NMO Diagnosis (IPND) has recently introduced new nomenclature and new diagnostic criteria based on the presence of AQP4 antibodies in patients’ serum [25]. The new nomenclature unifies all NMO-related diseases into NMOSD, further divided into NMOSD with AQP4 antibodies, NMOSD without anti-AQP4 antibodies and NMOSD with unknown AQP4 antibody status. These new criteria led to an increase in the number of clinical conditions that require testing for anti-AQP4 antibodies [26].

Thus, testing for anti-AQP4 antibodies is important not only for early and accurate diagnosis but also for treatment strategies, since these differ considerably between NMO and MS. Indeed, while immunomodulatory drugs such as β-interferons and glatiramer acetate are believed to be preferential in MS, immunosuppressive drugs and in particular B cell targeting therapies are thought to be favorable in the treatment of NMO.

Several assays have been developed to detect antibodies to AQP4. Serological assays based on the use of AQP4-M1 isoform were the first that were used [10, 27–30]. Only recently, following the demonstration that OAPs are the main target of NMO-IgG [31] has AQP4-M23 become more widely used as the target antigen [29].

Using an in-house ELISA assay, Kim et al. [16] tested both isoforms in parallel and although they did not find any difference between isoforms in the detection rate among definite or high-risk NMO patients, they chose to use the M23 isoform, because it increased the signal-to-noise ratio. Similarly, by commercial CBA, Waters et al. found no difference in sensitivity when comparing the human M1 or M23 isoforms, reporting, however, an increase in signal using the M23. In contrast, Mader et al. [29] using live human embryonic kidney (HEK) cells, noted a higher sensitivity of the M3 isoform. These authors also observed a different staining pattern in the two assays. Antibody binding to the M1 isoform showed much smaller punctate staining compared with the M23 isoform, supporting previous freeze fracture data showing that the M23 isoform forms OAP in HEK cells [29].

In addition, using sera of patients with NMO and recombinant AQP4-specific monoclonal antibodies, Crane et al. [32] showed that AQP4-IgG monoclonal antibodies or their Fab fragments had consistently greater affinity for M23 than for M1 because of OAP assembly, again suggesting that the choice of AQP4 isoforms, and therefore OAP assembly, can directly influence assay sensitivity.

The aim of the present study was to evaluate the performance of a new commercially available ELISA using the M23 antigen to detect anti-AQP4 antibodies. Results were compared with those obtained with a multiparametric commercial IIF assay. Although not directly compared with the ELISA using M1-AQP4, our data show a sensitivity (83.3%) that is higher than that reported in previous studies using the M1 isoform [17–19], similar to the mean sensitivity obtained with CBA (76.7%; range, 55.6–96.7%) and significantly higher than that of the other assays (range, 48.5–62.3%) [14].

Finally, ELISA assay has provided concordant results with multiparametric commercial IIF assay (100% concordance), which is currently the reference method to detect anti-AQP4 antibodies [33].

Our study also aimed to evaluate the analytical accuracy of this new anti-AQP4 ELISA assay. This is an important issue, because some methods have yielded discordant results when applied at different laboratories, and incongruous results were found with identical sample tested in different assays [34]. However, reproducible assays for the detection of anti-AQP4 antibodies are crucial for large multi-centre studies aiming to better define the epidemiological, clinical, and pathological features of patients with NMO and their response to treatment [35].

We found that the analytical imprecision, expressed as within-run, between-day and within-laboratory CV using two different antibody concentrations was low and that the total repeatability measured on the positive controls at low and high concentrations of AQP4-antibodies was even better than that stated by the manufacturer for the same concentration levels.

The good analytical precision and accuracy of the test and the availability of a fully automated system featuring relative ease of use and rapid response, lead us to conclude that the test is satisfactory for routine use and potentially suitable for long-term AQP4 antibody monitoring.

The step forward of the present study resides in two important aspects. One is the possibility of easy access for a wide range of laboratories to a simple quantitative anti-AQP4 ELISA test. The other is the demonstration of the absolute specificity and of the increased sensitivity of this new ELISA, similar to the current gold standard CBA method.

In conclusion, this study represents the first report evaluating the latest commercial ELISA method to detect anti-AQP4 antibodies, representing a valid alternative to CBA. We believe that our findings may lay the foundations to harmonize anti-AQP4 antibody detection, therefore facilitating earlier and more accurate diagnosis of NMOSD patients.
